# Supplementary material for: Based on Tau PET Radiomics Analysis for the Classification of Alzheimer’s Disease and Mild Cognitive Impairment
Source: Brain Sci. 2023 Feb 20;13(2):367. doi: 10.3390/brainsci13020367 (PMC9953966; doi:10.3390/brainsci13020367)
Supplement: Supplementary file 1 [file brainsci-13-00367-s001.zip › brainsci-2158499-supplementary.pdf]

Table S1. 60 ROIs with significant differences between AD and NC from ANDI training datasets on AAL atlas.

Brain region(AAL)	Voxels size	Cluster MNI k		
		X (mm)	Y (mm)	Z (mm)
**Temporal_Mid_L**	**4414**	**-6**	**-22**	**-54**
**Temporal_Mid_R**	**3642**	**-6**	**-22**	**-54**
**Frontal_Mid_R**	**3417**	**-6**	**-22**	**-54**
**Frontal_Mid_L**	**3366**	**-6**	**-22**	**-54**
**Occipital_Mid_L**	**3157**	**-6**	**-22**	**-54**
**Temporal_Inf_R**	**3130**	**-6**	**-22**	**-54**
**Temporal_Inf_L**	**3100**	**-6**	**-22**	**-54**
**Precuneus_L**	**2795**	**-6**	**-22**	**-54**
**Precuneus_R**	**2702**	**-6**	**-22**	**-54**
**Fusiform_R**	**2422**	**-6**	**-22**	**-54**
**Frontal_Sup_R**	**2335**	**-6**	**-22**	**-54**
**Fusiform_L**	**2208**	**-6**	**-22**	**-54**
**Parietal_Inf_L**	**2107**	**-6**	**-22**	**-54**
**Temporal_Sup_R**	**2057**	**-6**	**-22**	**-54**
**Temporal_Sup_L**	**2043**	**-6**	**-22**	**-54**
**Frontal_Sup_L**	**2031**	**-6**	**-22**	**-54**
**Lingual_R**	**1974**	**-6**	**-22**	**-54**
**Lingual_L**	**1875**	**-6**	**-22**	**-54**
**Occipital_Mid_R**	**1778**	**-6**	**-22**	**-54**
**Frontal_Inf_Tri_L**	**1654**	**-6**	**-22**	**-54**
**Calcarine_L**	**1596**	**-6**	**-22**	**-54**
**Precentral_L**	**1483**	**-6**	**-22**	**-54**
**SupraMarginal_R**	**1469**	**-6**	**-22**	**-54**
**Angular_R**	**1368**	**-6**	**-22**	**-54**
**Angular_L**	**1350**	**-6**	**-22**	**-54**

Parietal_Sup_L	1348	-6	-22	-54
Calcarine_R	1314	-6	-22	-54
Cuneus_R	1312	-6	-22	-54
Cuneus_L	1305	-6	-22	-54
Frontal_Inf_Tri_R	1250	-6	-22	-54
Occipital_Sup_R	1189	-6	-22	-54
Parietal_Sup_R	1096	-6	-22	-54
SupraMarginal_L	1070	-6	-22	-54
Occipital_Sup_L	1044	-6	-22	-54
Parietal_Inf_R	990	-6	-22	-54
Postcentral_L	982	-6	-22	-54
Postcentral_R	977	-6	-22	-54
Insula_R	960	-6	-22	-54
Insula_L	958	-6	-22	-54
Cingulum_Ant_L	945	-6	-22	-54
Cingulum_Ant_R	936	-6	-22	-54
Cingulum_Mid_R	929	-6	-22	-54
Cingulum_Mid_L	924	-6	-22	-54
Frontal_Inf_Oper_R	923	-6	-22	-54
Frontal_Inf_Orb_L	844	-6	-22	-54
Precentral_R	838	-6	-22	-54
Supp_Motor_Area_R	792	-6	-22	-54
Frontal_Inf_Orb_R	753	-6	-22	-54
Frontal_Inf_Oper_L	696	-6	-22	-54
Supp_Motor_Area_L	649	-6	-22	-54
Frontal_Med_Orb_R	599	-6	-22	-54
Frontal_Mid_Orb_L	560	-6	-22	-54
Temporal_Pole_Sup_L	558	-6	-22	-54
Temporal_Pole_Sup_R	549	-6	-22	-54

**Rolandic_Oper_L**	**522**	**-6**	**-22**	**-54**
**Rolandic_Oper_R**	**513**	**-6**	**-22**	**-54**
**Caudate_L**	**760**	**-12**	**-6**	**-8**
**Caudate_R**	**574**	**-12**	**-6**	**-8**
**Thalamus_L**	**565**	**-12**	**-6**	**-8**
**Thalamus_R**	**515**	**-12**	**-6**	**-8**

Table S2.Selected features from ADNI cohorts.

**Brain region(AAL)**	**Global features**	**Texture features**			
		**GLCM**	**GLRLM**	**GLSZM**	**NGTDM**
**Cingulum_Mid_L**		**Contrast**		**LZE**	**Complexity,Contrast**
**Occipital_Mid_L**				**LZE**	
**Occipital_Mid_R**		**Entropy, Dissimilarity**		**LZE, GLN**	**Complexity**
**Fusiform_L**				**GLN**	
**Fusiform_R**		**Entropy**	**SRE**	**GLN**	
**Parietal_Inf_L**					**Complexity**
**Angular_R**				**GLN**	
**Temporal_Sup_L**				**GLN, ZP**	
**Temporal_Sup_R**				**LZE, GLN**	
**Temporal_Pole_Sup_R**	**Kurtosis**			**LZE**	**Complexity**
**Temporal_Inf_L**				**LZE, GLN**	**Complexity**
**Temporal_Inf_R**				**LZE, GLN**	**Contrast, Busyness, Complexity**

LZE: Large Zone Emphasis; GLN: Gray-Level Nonuniformity; SRE :Short Run Emphasis; ZP: Zone Percentage
